# Pemphigus vulgaris Treated With Rituximab: A Case Report

**DOI:** 10.7759/cureus.74511

**Published:** 2024-11-26

**Authors:** Guilherme N Faria, Pedro Fernandes, Diana Coelho, Susana Franco, Tiago Sardinha, Tiago Esteves

**Affiliations:** 1 Famiy Medicine, Centro de Saúde do Porto da Cruz, Serviço de Saúde da Região Autónoma da Madeira (SESARAM), Funchal, PRT; 2 Dermatology, Hospital Dr. Nélio Mendonça, Serviço de Saúde da Região Autónoma da Madeira (SESARAM), Funchal, PRT; 3 Family Medicine, Centro de Saúde de Santa Cruz, Serviço de Saúde da Região Autónoma da Madeira (SESARAM), Funchal, PRT; 4 Family Medicine, Centro de Saúde do Faial, Serviço de Saúde da Região Autónoma da Madeira (SESARAM), Funchal, PRT; 5 Family Medicine, Centro de Saúde do Caniço, Serviço de Saúde da Região Autónoma da Madeira (SESARAM), Funchal, PRT

**Keywords:** auto-imune diseases, biologic therapies, pemphigus in adults, pemphigus vulgaris, rituximab therapy

## Abstract

This case report highlights the efficacy of rituximab (RTX), a monoclonal antibody that targets B-lymphocytes, in the treatment of severe pemphigus vulgaris (PV) that was unresponsive to multiple conventional therapies. A 44-year-old female presented with mucocutaneous lesions that had been progressing for 12 months, with a Pemphigus Disease and Area Index (PDAI) total activity score of 66, indicating severe disease. The patient received two infusions of RTX two weeks apart, without complications. Complete remission of the lesions was observed after four months of follow-up. The successful outcome of this case report reinforces RTX as a first-line treatment option in severe PV cases, although its feasibility should still be considered. It is important to note that RTX treatment may have limitations due to its high cost, potential for immunosuppression, and relatively high relapse rate after treatment cessation. This case report provides insights into the potential use of RTX in the treatment of moderate to severe PV and emphasizes the need for further research to evaluate its efficacy, safety, and long-term outcomes.

## Introduction

Pemphigus is a group of uncommon autoimmune blistering disorders that result in intraepithelial blisters in mucous membranes and skin due to acantholysis, the loss of adhesion between keratinocytes. This process occurs because IgG autoantibodies target structural proteins in epidermal desmosomes that are essential for tissue integrity [1].

Pemphigus vulgaris and Pemphigus foliaceus are the two major variants, accounting for 90-95% of diagnoses. Pemphigus vulgaris (PV) typically begins in oral cavity lesions. Skin lesions in the cutaneous variant typically present as flaccid blisters, erosions, and crusts and can occur anywhere on the body. 

Diagnosis involves clinical presentation, serology, direct immunofluorescence microscopy of a perilesional biopsy, and histopathology of a lesional biopsy [2]. 

Corticosteroids are considered the mainstay of treatment. These are often combined with oral azathioprine or a mycophenolate compound to achieve disease control. In recent years, rituximab (RTX), an anti-CD20 immunobiological drug, has challenged the traditional treatment principles for pemphigus and is now being suggested as a first-line option, along with corticosteroids [3].

This case report aims to document the effectiveness and safety of using RTX as a treatment for a case of severe PV.

## Case presentation

**Figure 1:**
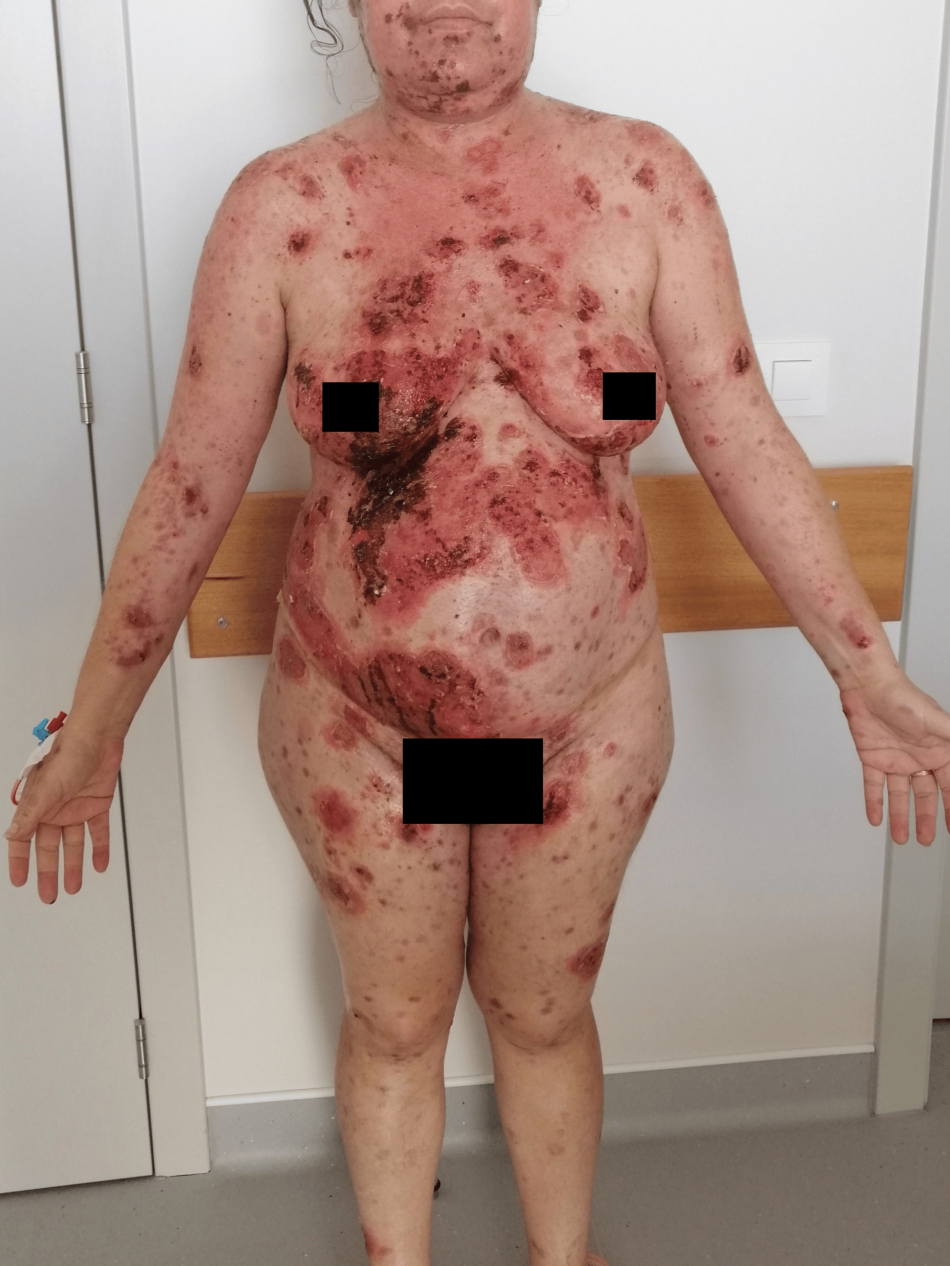
Dispersed flaccid blisters and erosions on the skin, mainly on the trunk, upper limbs, and face (anterior view).

**Figure 2:**
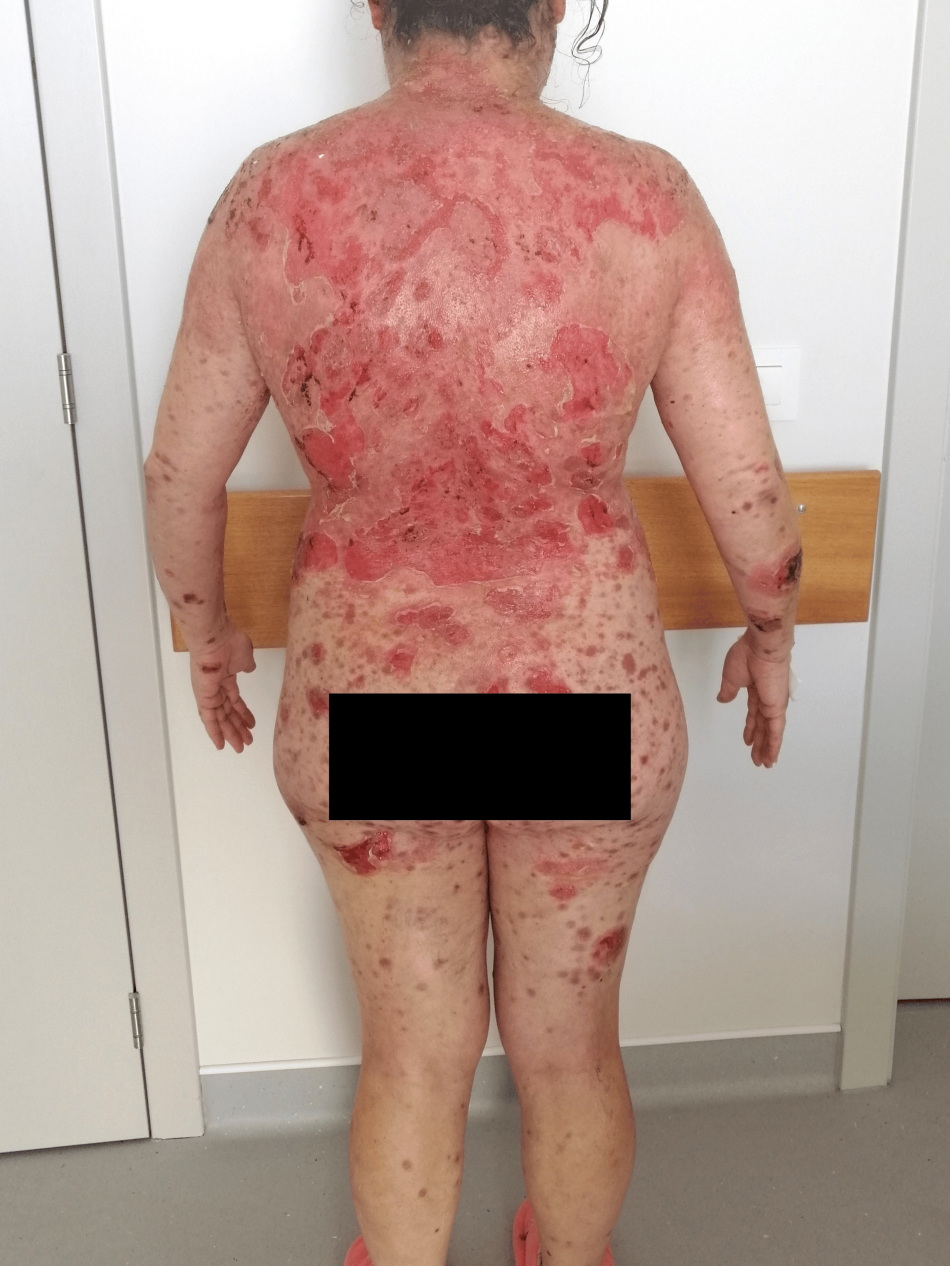
Dispersed flaccid blisters and erosions on the skin, mainly on the trunk, upper limbs, and face (posterior view).

**Figure 3:**
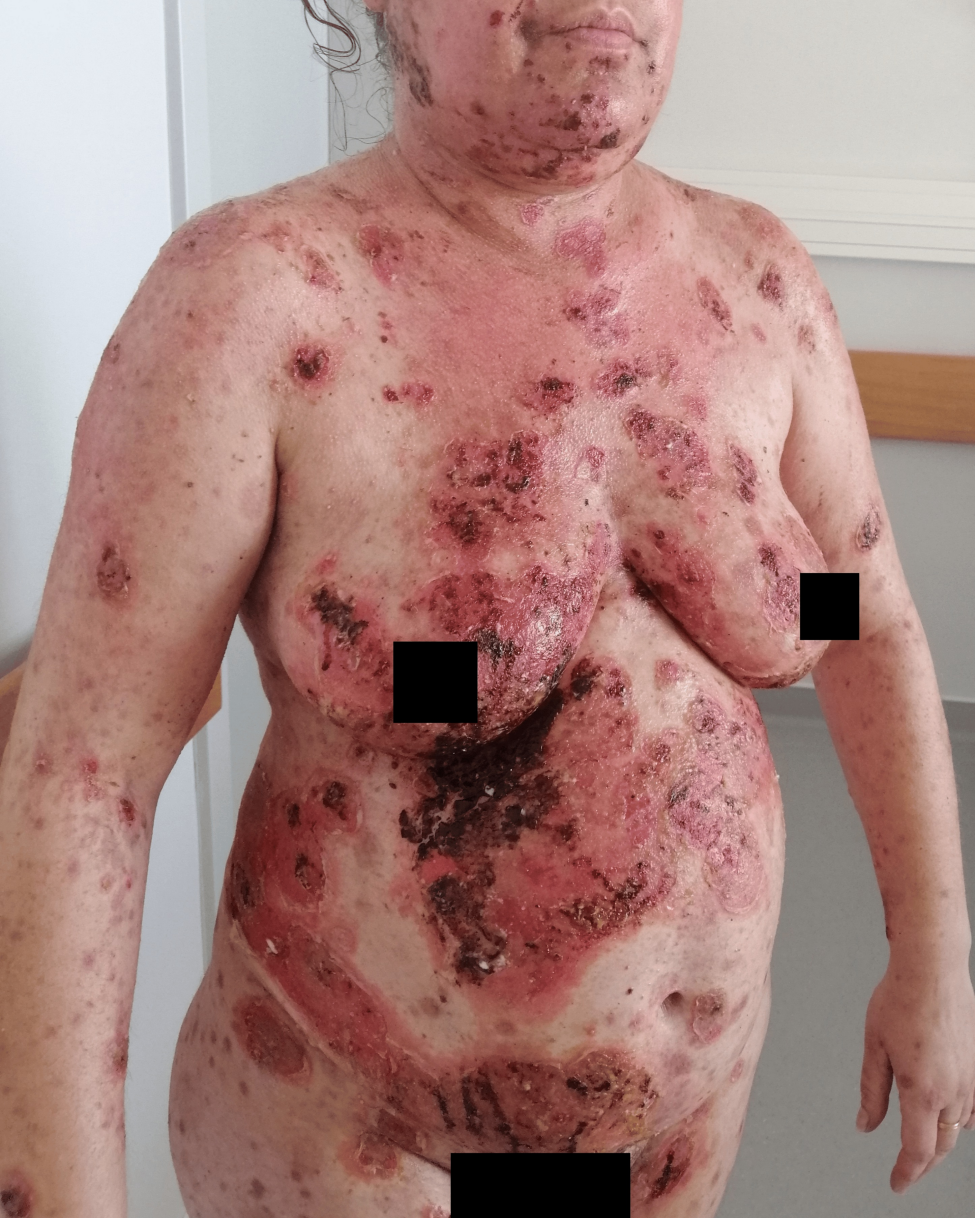
Dispersed flaccid blisters and erosions on the skin, mainly on the trunk, upper limbs and face (anterolateral view).

A 44-year-old female with no relevant risk factors or potential comorbidities presented with mucous and skin lesions that had been gradually evolving over 12 months, causing pain and a burning sensation. The patient had dispersed flaccid blisters and erosions on the skin, mainly on the trunk, upper limbs, and face, and several mucosal areas were affected, including the oral cavity, nasopharynx, and genital region (Figures 1-3). The patient had previously received outpatient treatment with oral prednisolone (60 mg/day), cyclosporine (400 mg/day), azathioprine (150 mg/day), and methotrexate (20 mg/day), without improvement. On admission, the patient had mild leukocytosis with neutrophilia, but no other relevant analytical changes were observed. A punch biopsy of a blister portion of the skin showed intraepidermal/suprabasal blistering and dyskeratotic cells with acantholysis, leading to a presumptive diagnosis of PV with mucocutaneous involvement with a Pemphigus Disease and Area Index (PDAI) total activity score of 66 [4], indicating severe disease.

**Figure 4:**
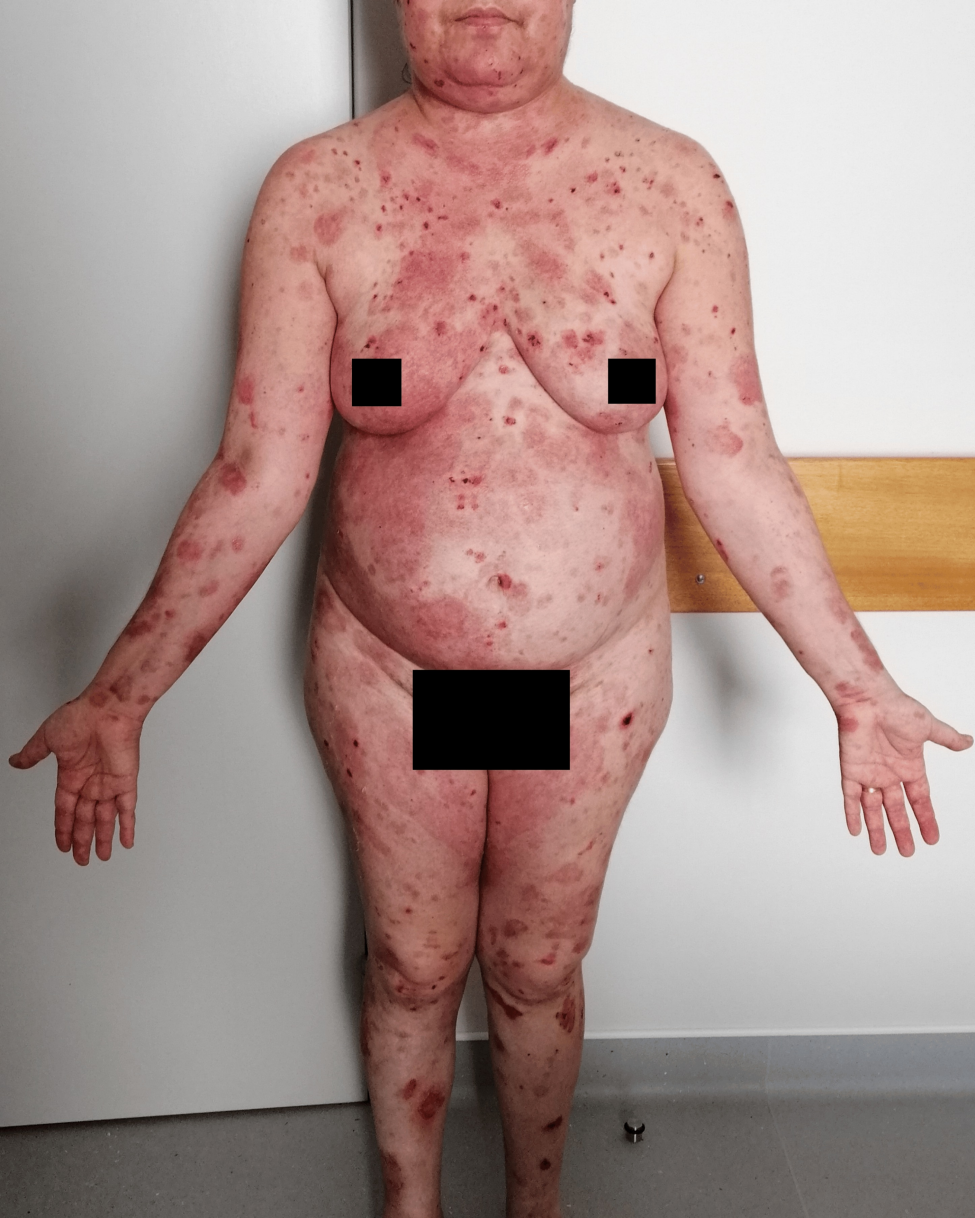
Partial remission of the lesions one week after the second administration of rituximab (anterior view).

**Figure 5:**
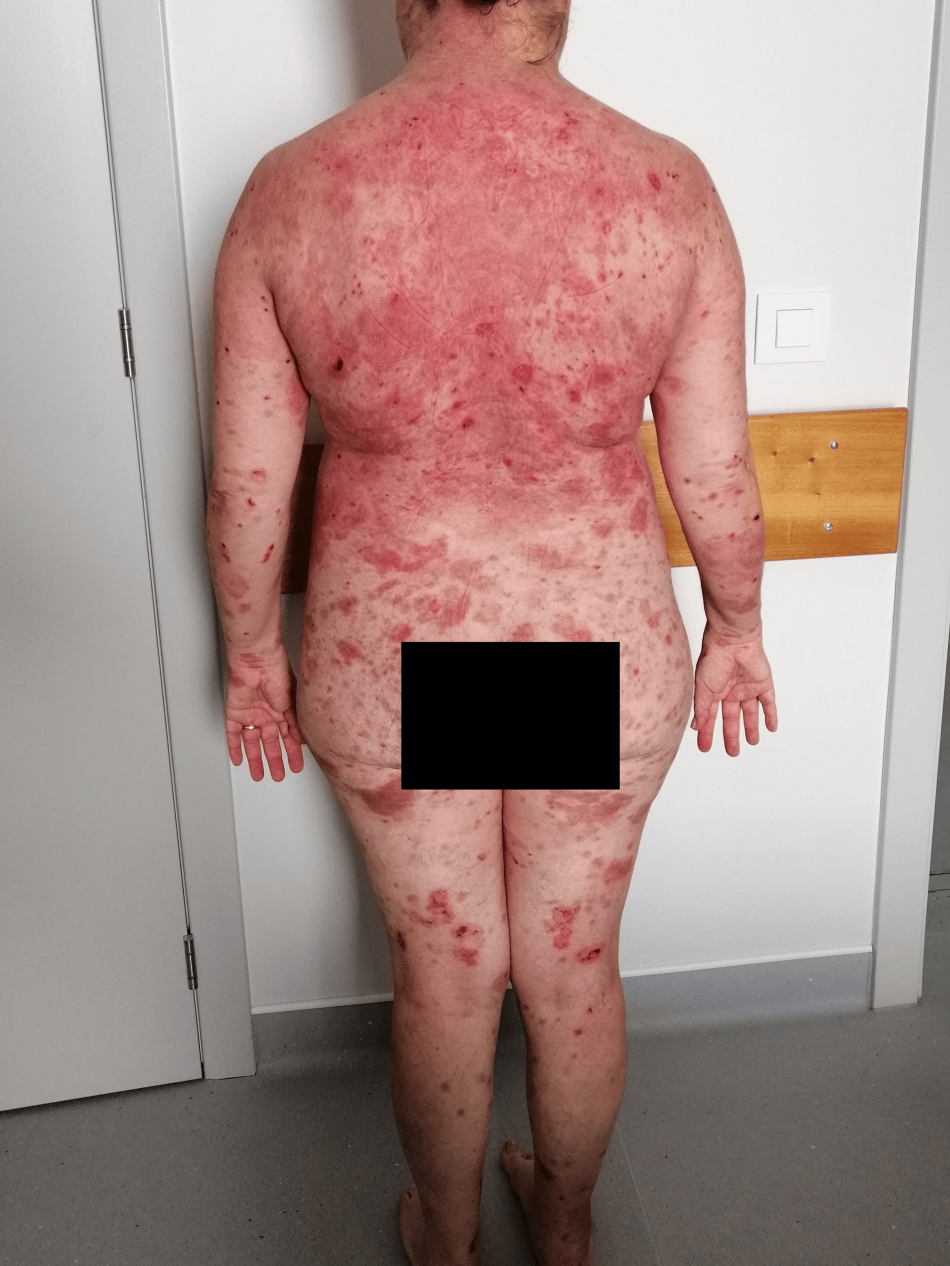
Partial remission of the lesions one week after the second administration of rituximab (posterior view).

**Figure 6:**
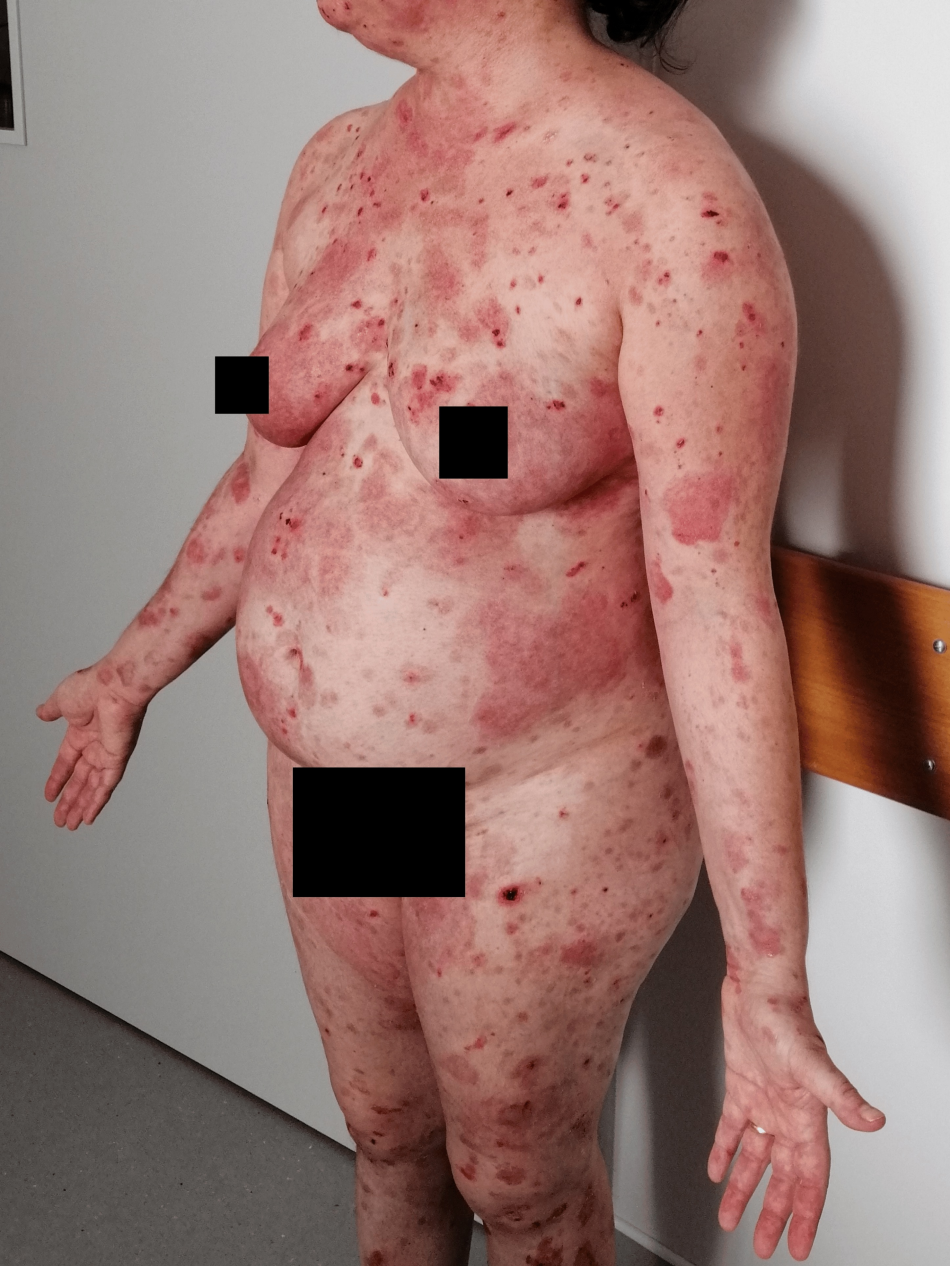
Partial remission of the lesions one week after the second administration of rituximab (anterolateral view).

**Figure 7:**
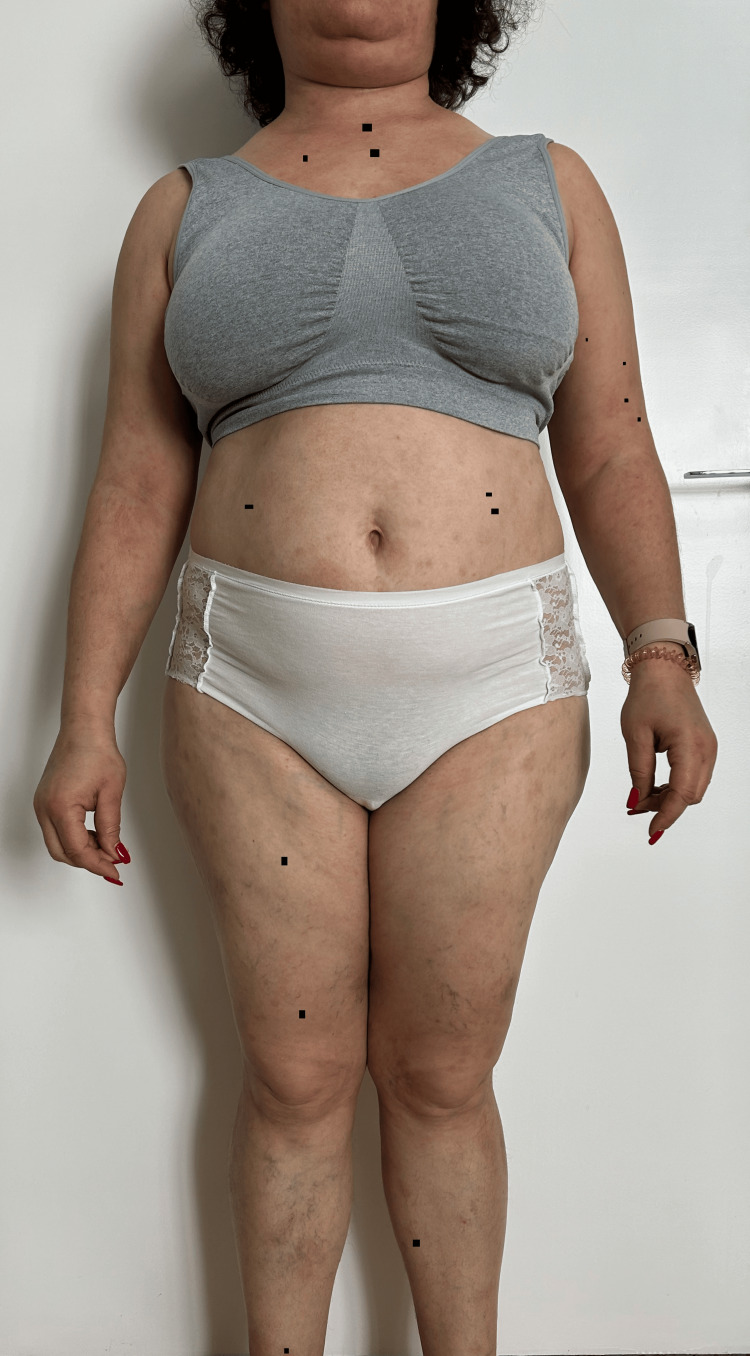
Remission of the lesions one week after maintenance treatment with rituximab at 12 months (anterior view).

**Figure 8:**
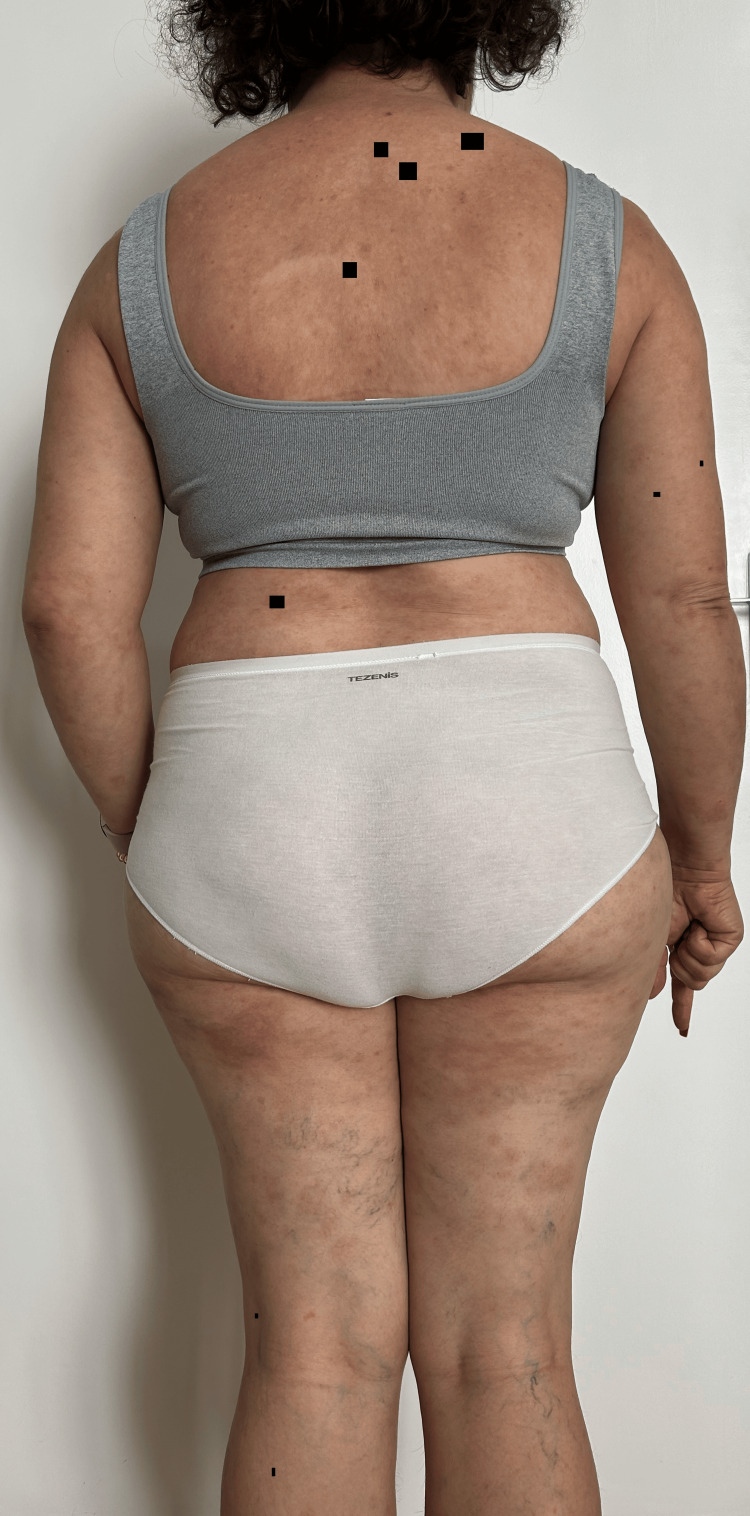
Remission of the lesions one week after maintenance treatment with rituximab at 12 months (posterior view).

**Figure 9:**
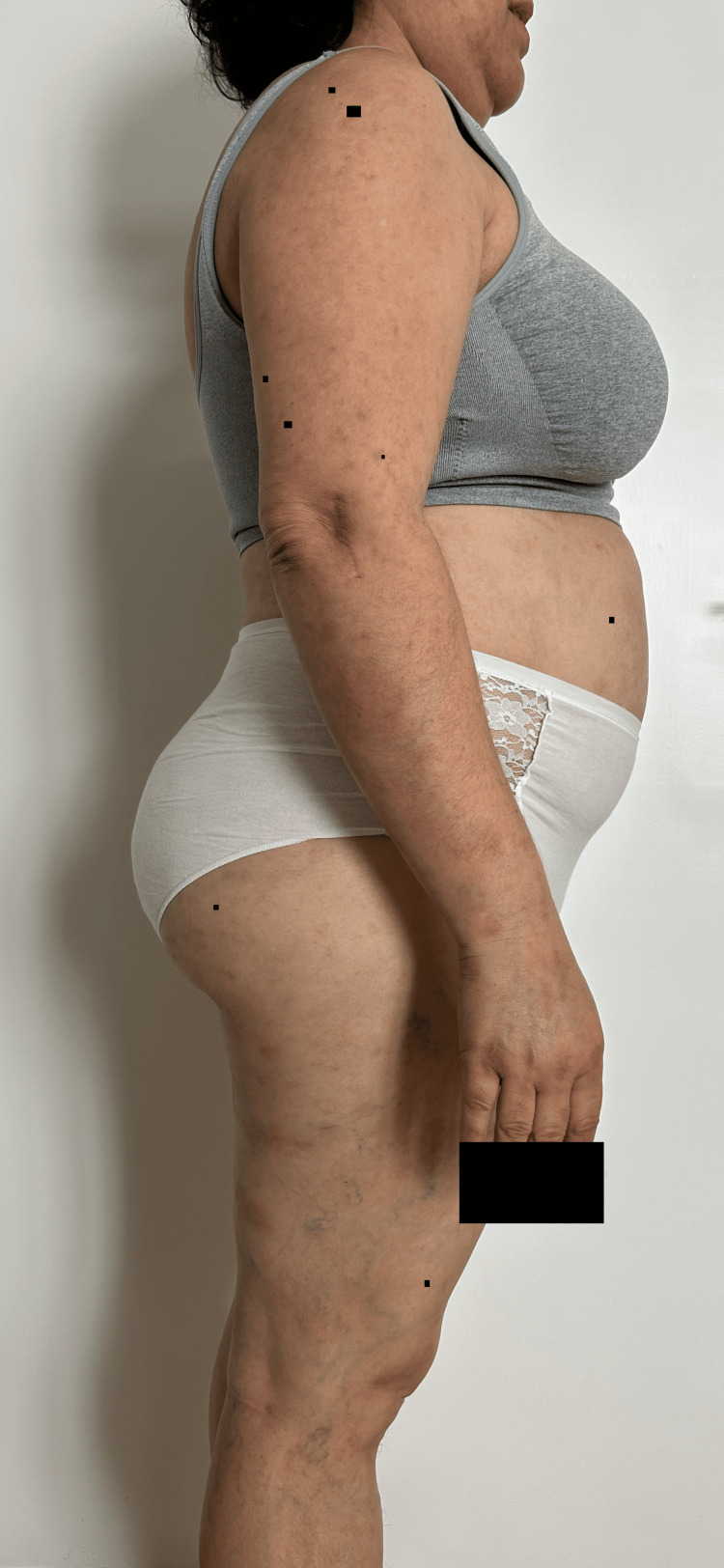
Remission of the lesions one week after maintenance treatment with rituximab at 12 months (lateral view).

Due to the patient's deteriorating condition, RTX infusions were recommended and administered in accordance with the European Academy of Dermatology and Venereology (EADV) guidelines for the management of pemphigus vulgaris and foliaceus [5] during a 30-day inpatient stay at the hospital-based dermatology service. The patient received two infusions of RTX 1g, two weeks apart, and oral prednisolone 60 mg/day, being closely monitored without complications. One week after the second administration of RTX, partial remission of the lesions was observed (Figures 4-6). Following two weeks of no new blister formation and an 80% improvement in the healing of established lesions, the corticosteroid dosage was gradually tapered. On an outpatient basis, remission was observed after four months of follow-up, presenting post-inflammatory hyperpigmentation dispersed on the skin as a result of resolving lesions. The patient has initiated maintenance treatment with RTX 500 mg at 12 months (Figures 7-9) and will continue receiving treatment at 18 months and every six months, if clinically indicated, according to the EADV guidelines on the management of pemphigus vulgaris and foliaceus.

## Discussion

The presented case demonstrates the successful treatment of severe PV with RTX after the failure of multiple conventional therapies. 

RTX is a monoclonal antibody that targets the CD20 antigen on B-lymphocytes and is effective in the treatment of PV. A meta-analysis of 30 studies including 578 patients with pemphigus reported a complete remission rate of 76% after one cycle of RTX. The mean time to remission was 5.8 months, and the median duration of response was 14.5 months [6]. A randomized controlled trial comparing RTX to conventional therapy (prednisolone alone) in 90 patients with newly diagnosed PV found that RTX induced remission in a significantly higher proportion of patients (89% vs. 34%) [7]. In addition, RTX was associated with a lower incidence of adverse events compared to conventional therapy.

Despite the promising results of RTX therapy in inducing remission in patients with PV, relapse can occur after treatment cessation. The overall relapse rate was reported as 40% in long-term follow-up. Studies suggest that higher doses of RTX may lead to longer remission durations, although both high- and low-dose regimens can be effective [6]. Furthermore, RTX therapy comes with limitations and challenges, such as its high cost and suppression of natural immunity by decreasing the levels of B-cells and the immunoglobulin IgM, which increases the risk of infection. Therefore, close monitoring is necessary for signs of infection during and after treatment to ensure prompt treatment if necessary. The optimal dose regimen and duration of RTX therapy for PV have not been fully established, and further studies are needed to address these issues. 

## Conclusions

The presented case highlights the effectiveness of RTX in the treatment of severe PV that is refractory to conventional therapy. This is consistent with previous studies that have demonstrated the superiority of RTX over conventional therapy. While there are some limitations and challenges associated with RTX therapy, it offers a promising alternative to conventional therapy for the management of PV. Further studies are needed to determine the optimal dosing regimen and duration of treatment and to assess the long-term safety and efficacy of RTX therapy in PV.
